# Glycoside Hydrolases from a targeted Compost Metagenome, activity-screening and functional characterization

**DOI:** 10.1186/1472-6750-12-38

**Published:** 2012-07-03

**Authors:** Michael J Dougherty, Patrik D’haeseleer, Terry C Hazen, Blake A Simmons, Paul D Adams, Masood Z Hadi

**Affiliations:** 1 The Joint BioEnergy Institute, Emeryville, USA; 2 Sandia National Laboratories, Livermore, USA; 3 Lawrence Livermore National Laboratory, Livermore, USA; 4 Physical Biosciences Division, Lawrence Berkeley National Laboratory, Berkeley, USA; 5 Department of Bioengineering, University of California, Berkeley, USA

## Abstract

**Background:**

Metagenomics approaches provide access to environmental genetic diversity for biotechnology applications, enabling the discovery of new enzymes and pathways for numerous catalytic processes. Discovery of new glycoside hydrolases with improved biocatalytic properties for the efficient conversion of lignocellulosic material to biofuels is a critical challenge in the development of economically viable routes from biomass to fuels and chemicals.

**Results:**

Twenty-two putative ORFs (open reading frames) were identified from a switchgrass-adapted compost community based on sequence homology to related gene families. These ORFs were expressed in *E. coli* and assayed for predicted activities. Seven of the ORFs were demonstrated to encode active enzymes, encompassing five classes of hemicellulases. Four enzymes were over expressed *in vivo*, purified to homogeneity and subjected to detailed biochemical characterization. Their pH optima ranged between 5.5 - 7.5 and they exhibit moderate thermostability up to ~60-70°C.

**Conclusions:**

Seven active enzymes were identified from this set of ORFs comprising five different hemicellulose activities. These enzymes have been shown to have useful properties, such as moderate thermal stability and broad pH optima, and may serve as the starting points for future protein engineering towards the goal of developing efficient enzyme cocktails for biomass degradation under diverse process conditions.

## Background

The development of efficient routes to fuels and chemicals from lignocellulosic biomass is an area of significant research interest. Terrestrial lignocellulosic materials are composed of cellulose, hemicelluloses, and lignin. Cellulose, a crystalline homopolymer of glucose accounts for 40–50% of plant biomass, and as such is a potentially enormous source of glucose for microbial fermentation to valuable products [1]. Hemicelluloses, on the other hand, are heterogeneous polymers of various pentose and hexose sugars, accounting for 25–35% of biomass, and these sugars are a significant potential source of carbon and energy for biorefinery processes [2]. Currently the process to access these sugars requires a harsh chemical pre-treatment of plant biomass followed by digestion using a cocktail of secreted fungal enzymes to liberate sugars for fermentation. Despite the large amount of available plant material on a renewable and sustainable basis, the economical production of fermentable sugars from lignocellulosic biomass is currently hindered by several factors. These include the energy intensive pretreatment step, high production costs of biomass, and low catalytic efficiency of hydrolytic enzymes used for depolymerisation of cellulose. Identifying and/or engineering glycoside hydrolases (GHs) with improved enzymatic properties is a necessary step in reducing these costs [3].

It has been a long held desire to design efficient biocatalytic processes via amenable microbial activities. Environmental genomics, also known as metagenomics, has great potential for the discovery of new enzymes and pathways for biomass conversion [4]. Microorganisms from natural environments are a rich source of new biocatalysts and potentially conceal a massive trove of unknown encoded biocatalysts. The attempt to access these biocatalysts has been hindered by the ability to culture microbes in the laboratory. However, recent advances in DNA sequencing technology has made it possible to interrogate the sequence space of an organism or community to recover large numbers of putative coding regions in a high throughput manner without the need for laboratory culturing and propagation [5]. This approach allows for the detection of functional genes encoding biocatalysts of interest and has recently been successfully used for GH gene discovery using samples from the cow rumen microbial community [6] and in the microbial community associated with earthworm casts [7].

Functional metagenomics is typically performed through the analysis of ORFs using a functional assay and is currently the major bottleneck for biocatalyst discovery and validation. Careful consideration of the environment not only allows for discovery of appropriate biocatalysts but also increases the chances of identifying novel biocatalysts with unique structural and kinetic properties. To that end we interrogated a switchgrass-adapted compost microbial community [8] to identify genes predicted to encode enzymes with diverse activities on hemicelluloses, including endoxylanase, α-arabinofuranosidase, β-xylosidase, α-fucosidase, and acetyl xylan esterase. We then expressed these genes in *E. coli*, followed by purification and biochemical characterization of four of the resulting enzymes, focusing on properties important for the process of biomass hydrolysis. These enzymes may serve as useful scaffolds for protein engineering towards the goal of developing a suite of enzymes suitable for efficient hydrolysis of lignocellulosic biomass. This study validates the approach of targeted metagenomics for identifying potentially useful biocatalysts for specific process environments.

## Results

### Cloning 22 putative CAZy-like genes from the compost metagenome

**Figure 1:**
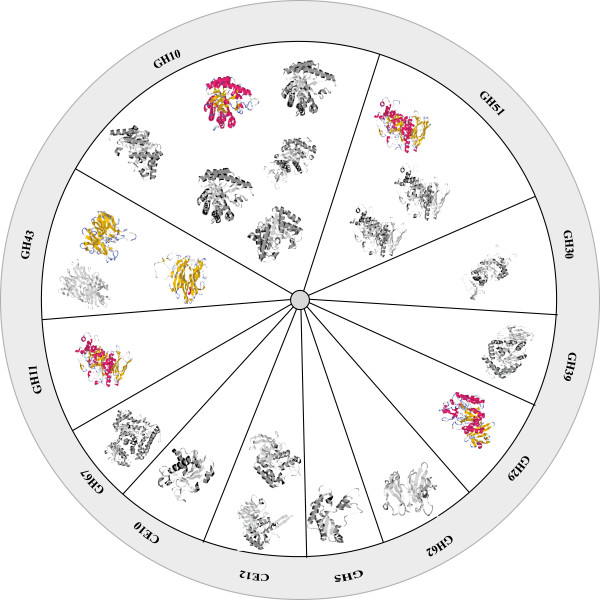
**Diverse CAZy-family genes members were identified in the metagenome from switchgrass compost.** The putative reconstructed genes are predicted to fall into 12 different CAZy families. The most abundant families are GH10, GH43, and GH51. Homology-based protein structure predictions were generated using AS2TS (proteinmodel.org) [10]. All members were expressed in E coli and screened for expression and activity. Colored structures were expressed and characterized biochemically while the black and white structures were not.

Sequence analysis of the respective compost metagenomic data identified 22 putative full-length hemicellulase sequences spanning various families in CAZy (Carbohydrate-Active enZymes database, http://www.cazy.org) (Figure 1) [9]. The 22 genes were codon-optimized for recombinant expression in *E. coli* and synthesized commercially (GenScript, Piscatawy, NJ). Each of the genes was PCR-amplified and cloned into pDONR221 to produce a Gateway entry clone. Three expression clones for each gene were generated from the entry clones using pET57-DEST, pET60-DEST, and pVP16 as destination vectors. The total set of expression clones incorporated a variety of promoters, solubility tags, and affinity tags. 

### Expression screening

**Figure 2:**
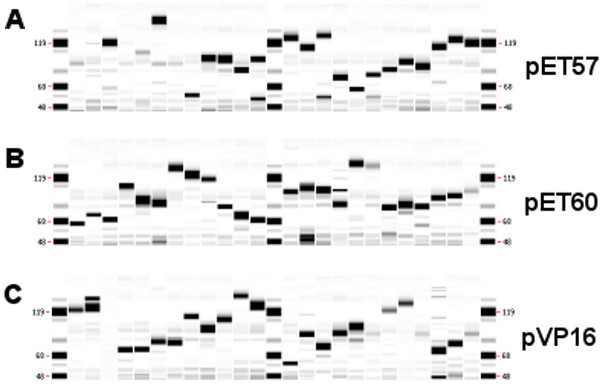
**All of the codon-optimized genes are highly expressed in E. coli.** Twenty-two genes in each of three expression vectors were expressed in *E. coli*. pET57 expression lysates (panel **A**), pET60 expression lysates (panel **B**), and pVP16 expression lysates (panel **C**) were analyzed with a LabChip GXII Protein Assay (Caliper Life Sciences, Hopkinton, MA). Ordering of samples for each panel is alphanumeric for each vector backbone. Expression of all genes was confirmed in each of the three vectors.

The expression clones were transformed into appropriate expression host cell lines (BL21 for the pVP16-derived clones, BL21(DE3) for the pET-derived clones). Transformants were selected and grown for protein expression in 96-deep-well plates in auto-induction medium [11]. Cells were harvested by centrifugation, lysed using lysozyme and Triton X-100, and clarified by centrifugation. Soluble and insoluble fractions were analyzed on a Caliper GXII microfluidics platform with LIMS integration for automated QC of protein expression and solubility (Figure 2). We were able to achieve expression of all of the genes in each of the three expression vectors, as judged by analysis of the cell lysates. Western blot analysis with anti-His tag antibodies detected varying levels of soluble protein expression for every ORF in each of the three vectors (data not shown). Significant soluble expression was achieved for 10 of 22 genes in pET57, 4 of 22 in pET60, and 9 of 22 in pVP16. 

### Activity screening

The entire set of soluble lysates was tested for endoxylanase, β-xylosidase, and α-arabinofuranosidase activities via colorimetric assays (Table 1). Specific lysates were also tested for α-glucuronidase, cellulase, α-fucosidase, β-glucosidase, and acetyl esterase activities. In total, we were able to confirm seven of the predicted activities from twenty-two ORFs assayed. Four of the enzymes with confirmed activities were purified to homogeneity and characterized in detail.

**Table 1:** Activity screening of putative CAZy-related genes

**ORF**	**Family**	**Predicted activity**	**Active in screen**
JMC02101a	GH5	cellulase	No
JMC03259	GH10	endoxylanase	No
JMC14824	GH10	endoxylanase	No
JMC04931	GH10	endoxylanase	No
JMC07159	GH10	endoxylanase	No
JMC07447	GH10	endoxylanase	No
JMC37744	GH10	endoxylanase	Yes
JMC01245	GH11	endoxylanase	Yes
JMC09349	GH29	α-fucosidase	Yes
JMC02101b	GH30	β-glucosidase	No
JMC44805	GH39	β-xylosidase	No
JMC25406	GH43	β-xylosidase/α-arabinofuranosidase	Yes
JMC04168	GH43	β-xylosidase/α-arabinofuranosidase	Yes
JMC35591	GH43	β-xylosidase	No
JMC02364	GH51	α-arabinofuranosidase	No
JMC00766	GH51	α-arabinofuranosidase	No
JMC15193	GH51	α-arabinofuranosidase	Yes
JMC04920	GH62	α-arabinofuranosidase	No
JMC10050	GH67	α-glucuronidase	No
JMC04701	CE10	pectin acetylesterase	No
JMC16911	CE12	acetyl xylan esterase	Yes
JMC10774	CE12	acetyl xylan esterase	No

Twenty-two ORFs from 12 CAZy families were screened for expression and activity. All of the ORFs could be expressed in soluble form in *E. coli* and seven of twenty-two ORFs demonstrated the predicted activity.

### Purification and characterization of JMC25406

**Figure 3:**
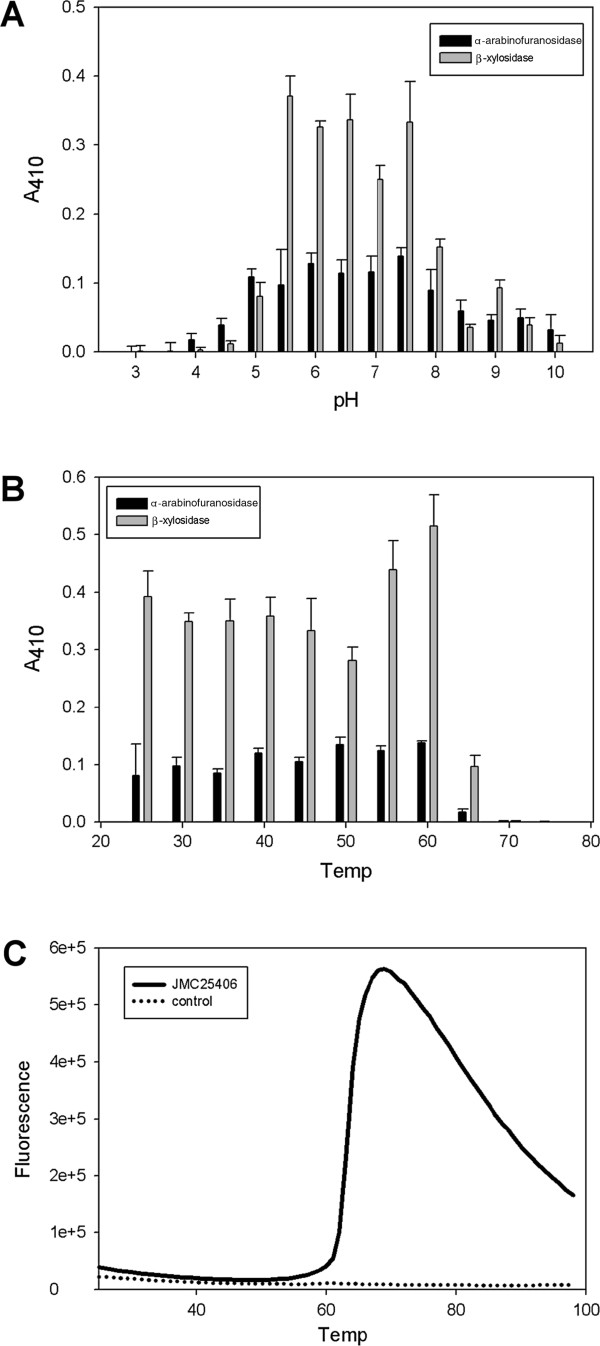
**Purified JMC25406 is active at neutral pH and is moderately thermostable.A** – JMC25406 has a pH optimum between 5.5 and 7.5 for both α-arabinofuranosidase and β-xylosidase activities. **B** – JMC25406 enzymatic activities are stable to heating up to 60°C for 20 min. **C** – DSF of purified JMC25406 gives an estimated T_M_ of 65.6°C. A representative plot from triplicate experiments is shown for purified JMC25406 and a buffer control.

JMC25406, predicted to be a bi-functional β-xylosidase/α-arabinofuranosidase, was purified (>90%) by affinity chromatography making use of the encoded fusion with His and GST tags. The GST tag was readily cleaved by TEV protease and a final polishing step was performed on Q anion exchange resin. The purified protein had a specific activity of 14.3 ± 0.6 U/mg using 4-nitrophenyl β-d-xylopyranoside at pH 7.5 and 7.6 ± 0.2 U/mg using 4-nitrophenyl α-L-arabinofuranoside at pH 5.5. These values are comparable to values from previous literature reports for enzymes from this family [9]. Interestingly JMC25406 showed a broad pH optimum for both activities ranging between pH 5.5-7.5 (Figure 3A). The enzyme was stable to heating up to 60°C for 20 min, but lost activity rapidly above that temperature (Figure 3B). We then performed additional experiments that showed that JMC25406 maintained ~75% of its activity after 16 hours at 60°C (data not shown). These data agree well with the results of differential scanning fluorimetry (DSF), which estimated the T_M_ of the protein to be 65.6°C (Figure 3C). 

### Purification and characterization of JMC01245 and JMC37744

**Figure 4:**
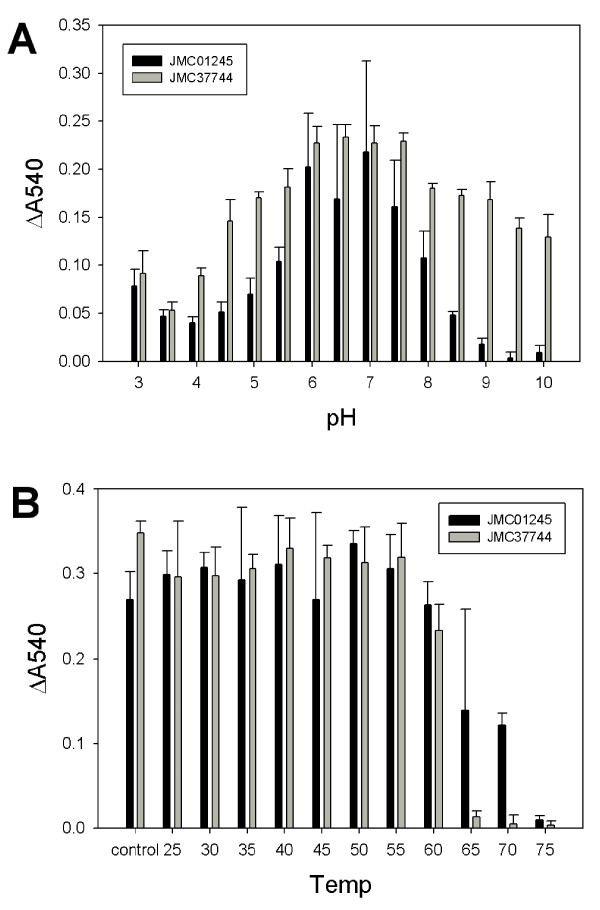
**JMC01245 and JMC37744 have broad pH optima and moderate thermal stability.A** – Both JMC01245 and JMC37744 have pH optima for endoxylanase activity around 6–7, but JMC37744 maintains significant activity up to pH 10. **B** – JMC37744 endoxylanase activity is resistant to heating up to 60°C, while JMC01245 activity is stable up to 70°C.

JMC01245 and JMC37744, predicted to be endoxylanases, were purified (>75%) using affinity chromatography making use of the fusion with His and MBP tags. TEV cleavage of JMC01245 was efficient, but once cleaved the free protein was prone to aggregation, while the MBP-JMC37744 fusion protein was a poor substrate for TEV protease, possibly due to inaccessibility of the cleavage recognition site in the folded fusion protein. Both proteins were characterized as fusion proteins with appropriate negative controls. Both enzymes showed endoxylanase activity on wheat arabinoxylan, with specific activities of 7.2 (JMC01245) and 4.4 (JMC37744) U/mg, and had pH optima around 6–7 (Figure 4A). These properties were similar to those of commercially available enzymes. Interestingly, JMC37744 showed greater activity than JMC01245 at higher pH, maintaining >50% of maximal activity at pH 10. Both enzymes were moderately thermostable, with JMC37744 maintaining ~75% of its maximal activity up to 60°C, and JMC01245 maintaining ~40% of maximal activity up to 70°C (Figure 4B).

### Purification and characterization of JMC09349

**Figure 5:**
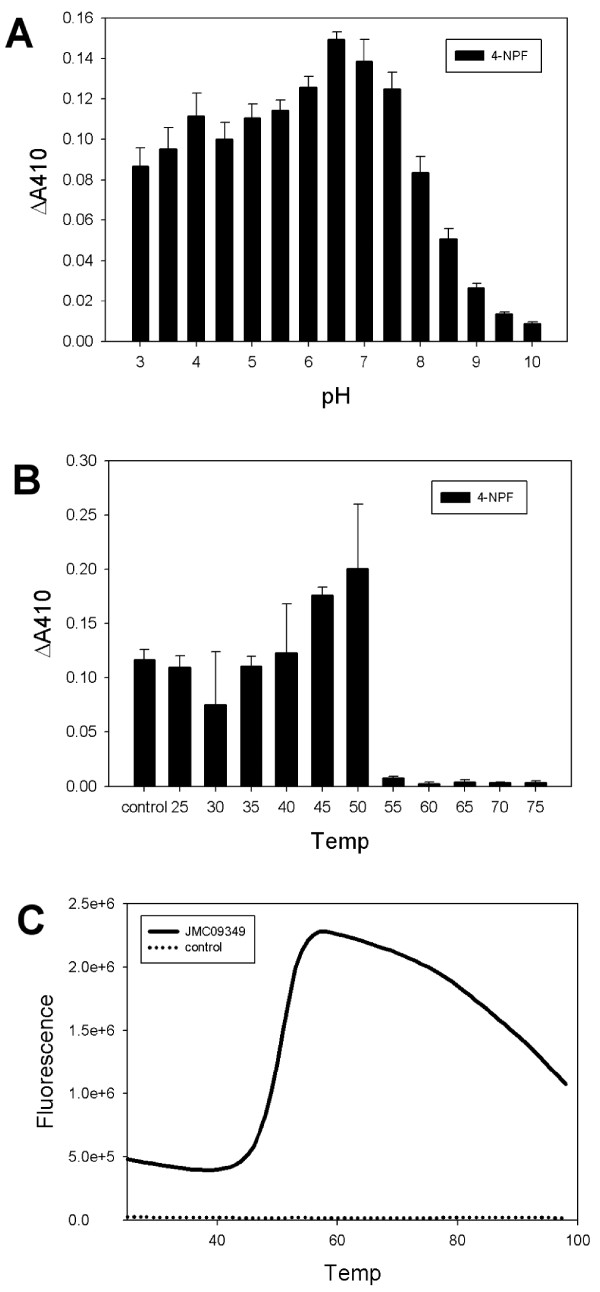
**JMC09349 has a broad pH optimum and moderate thermal stability.A** – JMC09349 has a pH optimum of 6.5 and maintains significant α-fucosidase activity between pH 3–8.5. **B** – JMC09349 enzymatic activity is thermo-tolerant up to 50°C for 20 min. **C** – DSF of purified JMC09349 gives an estimated T_M_ of 50.3°C. A representative plot from triplicate experiments is shown for purified JMC09349 and a buffer control.

JMC09349, predicted to be an α-fucosidase, was purified (>80%) using affinity chromatography making use of the encoded fusion with His and MBP tags and a Q anion-exchange chromatography step. MBP tag cleavage by TEV protease was not very efficient and the intact fusion protein was used for further characterization with appropriate negative controls. The purified fusion protein was active on the model substrate 4-nitrophenyl pyranofucoside, with a specific activity of 0.43 U/mg. The pH optimum is 6.5, and the enzyme maintains >50% of maximal activity between pH 3–8.0 (Figure 5A). The enzyme was stable up to 50°C for 20 min, but lost activity above that temperature (Figure 5B), which agrees well with the DSF results, which estimated the T_M_ of the protein to be 50.3°C (Figure 5C).

## Discussion

Metagenomic analysis is an extremely powerful approach for investigating the microbial ecology of diverse environments, and a useful tool for accessing genetic diversity for applications in biotechnology [5,12]. This genetic diversity can serve as an important source for new biocatalysts with desired characteristics [13,14], as well as providing targets for structure-function analysis expanding our understanding of protein structural space [15,16]. Environments where biomass turnover rates are high at elevated temperatures, such as compost, are potentially an important source of targeted novel biocatalysts for lignocellulose degradation, one of the major barriers to economically viable biofuel production.

Our work in cloning, expressing, and assaying 22 putative CAZy enzymes has validated the targeted metagenomic approach for GH discovery by identifying 7 active hits from 22 screened ORFs. We used a high-throughput cloning approach using the Gateway system and liquid-handling robots for cloning the 22 ORFs into multiple expression vectors, expression screening, and activity screening. This approach has allowed us to rapidly identify vector and host combinations that result in well-behaved expression of ORFs.

Four of the expressed enzymes were purified and characterized in detail. JMC25406, predicted to be a GH43 family bifunctional β-xylosidase/α-arabinofuranosidase, showed robust activity of 7.6 and 14.3 U/mg protein on model substrates (using 4-nitrophenyl-α-L-arabinofuranoside and 4-nitrophenyl-β-d-xylopyranoside respectively) for both activities, comparable to literature values as well as commercially available standards. Compared to a previously described GH43 enzyme isolated from compost [17], JMC25406 exhibited similar pH profile (pH 5.5-7.5), but surprisingly increased thermal stability (by 10°C). Due to its dual functions, high thermal stability, and ease of purification, JMC25406 is an excellent starting point for further protein engineering for enzymatic cocktail development for various potential pretreatment strategies. JMC01245, a GH11 family protein, and JMC37744, a GH10 family protein, were demonstrated to possess the predicted endoxylanase activity. Both enzymes had similar properties; pH optima around 6.5 and stability to 60–70°C. JMC37744 displays activity over a very broad pH range (pH 5–10). JMC09349 was demonstrated to possess the predicted α-fucosidase activity, and has a broad pH optimum around 3–8.5 and modest thermostability up to 50°C. Taken together, the characterized enzymes pH optima and thermal stability profiles are quite consistent with the culture conditions of the compost community [8], further highlighting the importance of altering culture conditions in metagenomics experiments to enrich for enzymes with desired properties. These data indicate that it may be possible to isolate enzymes with robust activity in specific operating conditions (temperature, pH, solvents) by tailoring matching cultivation conditions and a recent publication on a similar compost community grown under thermophilic conditions found that culture supernatants were enriched for thermotolerant cellulose and hemicellulase activities [18].

Although metagenomic analysis is a powerful tool for enzyme discovery, the limiting factor for success using this approach remains time-consuming soluble protein expression and purification and biochemical characterization. The development of generalized methods for enzyme purification and assay with increased throughput will be necessary to fully harness the immense genetic diversity present in natural environments.

## Conclusions

Twenty-two putative CAZy ORFs were cloned into expression vectors and screened for soluble expression and activity using various glycoside hydrolase assays. Six ORFs were demonstrated to have the predicted activity when expressed in *E. coli*. Four of these active enzymes showing diverse hemicellulase activities were purified to near homogeneity, characterized biochemically, and shown to have broad pH optima and moderate thermostability.

## Methods

### Metagenome sequencing, assembly and analysis

Details of the sequence analysis and identification of the enzyme sequences have been described in detail previously [8]. Briefly, metagenomic DNA from a 31-day Switchgrass-Adapted Compost Community was shotgun sequenced using the Roche 454 GS FLX Titanium technology by the DOE Joint Genome Institute, and assembled using Newbler (version 2). Candidate full-length glycoside hydrolase enzyme sequences were identified by BLASTX hits (E < 1e^-10^) matching functionally characterized enymes in the CAZy database [9] over at least 90% of their length. Frameshift errors due to 454 homopolymer sequencing errors were identified and manually corrected based on the gapped BLASTX alignment to the closest protein sequences from NR.

### Materials and strains

All chemicals were purchased from Sigma-Aldrich (St. Louis, MO). β-xylanase M1, α-L-arabinofuranosidase, and exo-1,4-β-d-xylosidase were purchased from Megazyme (Wicklow, Ireland). Halt Protease Inhibitor was purchased from Thermo Scientific (Rockford, IL). Codon-optimized genes were synthesized by GenScript (Piscataway, NJ). Oligonucleotides were purchased from IDT (Coralville, IA). TOP10 cells (Invitrogen, Carlsbad, CA) were used for routine cloning applications, while BL21 and BL21(DE3) were used for protein expression (Novagen, Madison, WI).

### Cloning

PCR was used to amplify the coding sequence of each ORF from pUC57-derived plasmid and append *attB* recombination sequences to each ORF, in order to create substrates for the Gateway cloning system (Invitrogen, Carlsbad, CA). To generate expression clones, an entry clone was constructed via BP Clonase reaction using purified PCR product and plasmid pDONR221. Expression clones were generated via LR Clonase reaction using pDONR221 derivatives and recipient plasmids pET57-DEST, pET60-DEST, and pVP16 according to manufacturer’s recommendations.

### Expression screening

*E. coli* expression hosts cells (BL21 for pVP16, BL21(DE3) for pET57-DEST and pET60-DEST) were transformed with appropriate expression plasmids and the resulting strains were grown in 2 mL of autoinduction medium [11] in 96 -well plates (catalog # 201379–100, Seahorse Bioscience, North Billerica, MA) for 16 hours at 30°C and 300 rpm. Cells were pelleted by centrifugation and lysed in 0.5 mL of buffer (50 mM Tris-Cl, pH 7.5, 100 mM NaCl, 1% Triton X-100, 0.1 mg/mL lysozyme, 2 mM MgCl_2_, 3 μg/mL DNase, 1X Halt Protease inhibitor). The resulting lysates were clarified by centrifugation (3200 x *g*), and after removal of the soluble material the insoluble pellet was solubilized with 4 M urea. The protein concentration of soluble and insoluble fractions was measured by Bradford assay and normalized. Protein analysis was performed using a capillary based instrument (LabChip GXII, Caliper Life Sciences, Hopkinton, MA) according to manufacturers recommendations. The GXII analyzes 96 proteins at a time with analysis time of ~ 40 sec per sample. The generated outputs were parsed and imported into our custom laboratory information database. The computed results were reviewed manually and validated.

### Activity screening

Endoxylanase activity was assayed at 40°C using 1% azo-wheat arabinoxylan as a substrate following the manufacturer’s protocol (Megazyme). β-xylosidase activity was assayed at 37°C and pH 7.5 in potassium phosphate buffer using 4-nitrophenyl β-d-xylopyranoside as the substrate [19]. α-arabinofuranosidase activity was assayed at 37°C and pH 5.5 in sodium acetate buffer using 4-nitrophenyl α-L-arabinofuranoside as the substrate. α-fucosidase activity was assayed at 37°C and pH 7.0 in HEPES buffer using 4-nitrophenyl pyranofucoside as the substrate. β-glucosidase was assayed at 37°C and pH 7.0 in HEPES buffer using 4-nitrophenyl glucoside as the substrate. Acetyl esterase was assayed at 25°C and pH 6.5 in MES buffer using 4-nitrophenyl acetate as the substrate. All PNP-based reactions were quenched by addition of an equal volume of 1 M sodium carbonate and absorbance at 410 nm (ε = 15,500 M ^-1^ cm^-1^) was measured. α-glucouronidase activity was assayed as described by Lee *et al*[20]. Cellulase activity was assayed at 37°C and pH 7.0 using carboxymethylcellulose as the substrate and the DNS assay for detection of reducing sugars [21]. Soluble fractions of cell lysates were used for the above assays.

### Purification and characterization of JMC25406

BL21 (DE3) was transformed with pET60-DEST-JMC25406 and grown in autoinduction medium at 30°C for 18–24 hours. Cells were harvested by centrifugation and stored at −20°C overnight. Cell pellets were resuspended (5 mL/g cell paste) in lysis buffer (50 mM sodium phosphate buffer, pH 8.0, 300 mM sodium chloride, 10 mM imidazole, 1 mg/mL lysozyme, 1X Halt protease cocktail inhibitor (Pierce)), incubated on ice for 20 min and lysed by sonication (Virsonic sonicator, medium tip, 8 × 10 s at power level 6). The lysate was clarified by centrifugation (30 min., 50,000 × *g*) and loaded onto a 1 mL HisTrap column (GE Healthcare), washed with lysis buffer, and eluted with a linear gradient from 10–500 mM imidazole in 50 mM sodium phosphate buffer, pH 8.0, 300 mM sodium chloride. The fusion protein eluted as a single major peak which was concentrated using Vivaspin concentrators (GE Healthcare), and buffer-exchanged into 50 mM Tris-Cl, pH 7.5. The sample was bound to immobilized glutathione resin (Pierce, Rockford, IL) and washed 3 times with 5 column volumes of buffer. On-column fusion partner cleavage was achieved by the addition of TEV protease (1:40 mass ratio) and incubated at 4°C for 18 hours. The released protein was loaded onto a HiTrap Q column and eluted with a linear gradient from 0–1 M NaCl in 50 mM Tris-Cl, pH 7.5. The protein was judged >90% pure by loading 2 μg of purified protein on SDS-PAGE gels and analysing the resulting image (data not shown). Specific activity of the purified enzyme was measured using the β-xylosidase and α-arabinofuranosidase assays as described above. The pH dependence of enzyme activity was determined using similar assays with various buffers (sodium citrate for pH 3.0-3.5, sodium acetate for pH 4.0-5.0, MES for pH 5.5-6.5, HEPES for pH 7.0-8.0, Bicine for pH 8.5, and CHES for pH 9.0-10.0). Thermal stability was analyzed by heating samples of JMC25406 diluted to 1.24 μM in a volume of 25 μL in 20 mM HEPES, pH 7.5 at various temperatures (25–75°C) for 20 min. The samples were then cooled to 4°C and assayed for β-xylosidase and α-arabinofuranosidase activities at pH 5.5 and 25°C. Differential scanning fluorimetry (DSF) was performed on samples (10 μM) of purified JMC25406 to determine the T_M_ of the protein. SYPRO Orange was used as the indicator dye; unfolding was monitored on an Applied Biosystems StepOne Plus Real-time PCR machine (Foster City, CA) heating from 25°C to 99°C at a rate of 1°C/min.

### Purification and characterization of JMC01245

BL21 was transformed with pVP16-JMC01245 and grown in autoinduction medium at 30°C for 18–24 hours. Cells were harvested by centrifugation and stored at −20°C overnight. Cell pellets were resuspended (5 mL/g cell paste) in lysis buffer (50 mM sodium phosphate buffer, pH 8.0, 300 mM sodium chloride, 10 mM imidazole, 1 mg/mL lysozyme, 1X Halt protease cocktail inhibitor (Pierce)), incubated on ice for 20 min and lysed by sonication (Virsonic sonicator, medium tip, 8 × 10 s at power level 6). The lysate was clarified by centrifugation (30 min., 50,000 × *g*) and loaded onto a 1 mL HisTrap column (GE Healthcare), washed with lysis buffer, and eluted with a linear gradient from 10–500 mM imidazole in 50 mM sodium phosphate buffer, pH 8.0, 300 mM sodium chloride. The fusion protein eluted as a single major peak (spanning several fractions) which was concentrated using Vivaspin concentrators (GE Healthcare), and buffer-exchanged into 20 mM Tris-Cl, pH 7.5, 300 mM NaCl. The protein was loaded onto a 5 mL MBPTrap column, washed with 20 mM Tris-Cl, pH 7.5, 300 mM NaCl, and eluted with the same buffer containing 10 mM maltose. The protein was judged ~75% pure by loading 2 μg of purified protein on SDS-PAGE gels and analysing the resulting image (data not shown). JMC01245 was assayed using 0.5% wheat arabinoxylan (low-viscosity, Megazyme) as the substrate and detection of reducing sugars with DNS. The pH dependence of enzyme activity was determined with various buffers (sodium citrate for pH 3.0-3.5, sodium acetate for pH 4.0-5.0, MES for pH 5.5-6.5, HEPES for pH 7.0-8.0, Bicine for pH 8.5, and CHES for pH 9.0-10.0). Thermal stability was analyzed by heating samples of JMC01245 diluted to 1.94 μM in a volume of 25 μL in 20 mM HEPES, pH 7.5 at various temperatures (25–75°C) for 20 min. The samples were then cooled to 4°C and assayed at pH 6.5 and 25°C.

### Purification and characterization of JMC37744

BL21 was transformed with pVP16-JMC37744 and grown in autoinduction medium at 30°C for 18–24 hours. Cells were harvested by centrifugation and stored at −20°C overnight. Cell pellets were resuspended (5 mL/g cell paste) in lysis buffer (50 mM sodium phosphate buffer, pH 8.0, 300 mM sodium chloride, 10 mM imidazole, 1 mg/mL lysozyme, 1X Halt protease cocktail inhibitor (Pierce)), incubated on ice for 20 min and lysed by sonication (Virsonic sonicator, medium tip, 8 × 10 s at power level 6). The lysate was clarified by centrifugation (30 min., 50,000  ×  *g*) and loaded onto a 1 mL HisTrap column (GE Healthcare), washed with lysis buffer, and eluted with a linear gradient from 10–500 mM imidazole 50 mM sodium phosphate buffer, pH 8.0, 300 mM sodium chloride. The fusion protein eluted as a single major peak (spanning several fractions) which were pooled, concentrated using Vivaspin concentrators (GE Healthcare), and buffer-exchanged into 20 mM Tris-Cl, pH 7.5, 300 mM NaCl. The protein was loaded onto a 5 mL MBPTrap column, washed with 20 mM Tris-Cl, pH 7.5, 300 mM NaCl, and eluted with the same buffer containing 10 mM maltose. The protein was judged ~80% pure by loading 2 μg of purified protein on SDS-PAGE gels and analysing the resulting image (data not shown). JMC37744 was assayed as described above for JMC01245. The pH dependence and thermal stability were also analyzed as described for JMC01245.

### Purification and characterization of JMC09349

BL21 was transformed with pVP16-JMC09349 and grown in autoinduction medium at 30°C for 18–24 hours. Cells were harvested by centrifugation and stored at −20°C overnight. Cell pellets were resuspended (5 mL/g cell paste) in lysis buffer (50 mM sodium phosphate buffer, pH 8.0, 500 mM sodium chloride, 20 mM imidazole, 1 mg/mL lysozyme, 1X Halt protease cocktail inhibitor (Pierce)), incubated on ice for 20 min and lysed by sonication (Virsonic sonicator, medium tip, 8 × 10 s at power level 6). The lysate was clarified by centrifugation (30 min., 50,000 × *g*) and loaded onto a 1 mL HisTrap column (GE Healthcare), washed with lysis buffer, and eluted with a linear gradient from 10–500 mM imidazole 50 mM sodium phosphate buffer, pH 8.0, 300 mM sodium chloride. The fusion protein eluted as a single major peak and concentrated using Vivaspin concentrators (GE Healthcare), and buffer-exchanged into 20 mM Tris-Cl, pH 7.5, 500 mM NaCl. The protein was loaded onto a 5 mL MBPTrap column, washed with 20 mM Tris-Cl, pH 7.5, 500 mM NaCl, and eluted with the same buffer containing 10 mM maltose. The MBPTrap eluate was diluted 40-fold in 20 mM Tris-Cl, pH 7.5, loaded onto a 1 mL HiTrap Q column and eluted with a linear gradient from 0.025-1 M NaCl in 20 mM Tris-Cl, pH 7.5. The protein was judged ~80% pure by loading 2 μg of purified protein on SDS-PAGE gels and analysing the resulting image (data not shown). The activity of the purified enzyme was measured using the α-fucosidase assay described above. The pH dependence of enzyme activity was determined using similar assays with various buffers (sodium citrate for pH 3.0-3.5, sodium acetate for pH 4.0-5.0, MES for pH 5.5-6.5, HEPES for pH 7.0-8.0, Bicine for pH 8.5, and CHES for pH 9.0-10.0). Thermal stability was analyzed by heating samples of JMC09349 diluted to 2 μM in a volume of 25 μL in 20 mM HEPES, pH 7.5 at various temperatures (25–75°C) for 20 min. The samples were then cooled to 4°C and assayed for α-fucosidase activity at pH 6.5 and 25°C. DSF was performed on samples of purified JMC09349 to determine the T_M_ of the protein as described for JMC25406.

## Competing interests

The authors declare that they have no competing interest.

## Authors’ contributions

MJD, PDH and MZH designed the research. MJD performed molecular biology and biochemical studies. PDH performed the bioinformatic analyses. MJD and MZH wrote the manuscript with substantial input from PDH, and PDA. All authors have read and approved the final manuscript.
